# Intraoperative cone beam computed tomography is as reliable as conventional computed tomography for identification of pedicle screw breach in thoracolumbar spine surgery

**DOI:** 10.1007/s00330-020-07315-5

**Published:** 2020-10-02

**Authors:** Gustav Burström, Paulina Cewe, Anastasios Charalampidis, Rami Nachabe, Michael Söderman, Paul Gerdhem, Adrian Elmi-Terander, Erik Edström

**Affiliations:** 1 Department of Clinical Neuroscience, Karolinska Institutet, Stockholm, Sweden; 2 Department of Neurosurgery, PO Neurokirurgi, Karolinska University Hospital, 171 64 Stockholm, Sweden; 3 Department of Trauma and Musculoskeletal Radiology, Karolinska University Hospital, Stockholm, Sweden; 4 Department of Clinical Sciences, Intervention and Technology (CLINTEC), Karolinska Institutet and Department of Reconstructive Orthopaedics, Karolinska University Hospital, Stockholm, Sweden; 5 Department of Image Guided Therapy Systems, Philips Healthcare, Best, The Netherlands; 6 Department of Neuroradiology, Karolinska University Hospital, Stockholm, Sweden

**Keywords:** Cone beam computed tomography, Image-guided surgery, Pedicle screws, Sensitivity and specificity

## Abstract

**Objectives:**

To test the hypothesis that intraoperative cone beam computed tomography (CBCT) using the Allura augmented reality surgical navigation (ARSN) system in a dedicated hybrid operating room (OR) matches computed tomography (CT) for identification of pedicle screw breach during spine surgery.

**Methods:**

Twenty patients treated with spinal fixation surgery (260 screws) underwent intraoperative CBCT as well as conventional postoperative CT scans (median 12 months after surgery) to identify and grade the degree of pedicle screw breach on both scan types, according to the Gertzbein grading scale. Blinded assessments were performed by three independent spine surgeons and the CT served as the standard of reference. Screws graded as Gertzbein 0 or 1 were considered clinically accurate while grades 2 or 3 were considered inaccurate. Sensitivity, specificity, and negative predictive value were the primary metrics of diagnostic performance.

**Results:**

For this patient group, the negative predictive value of an intraoperative CBCT to rule out pedicle screw breach was 99.6% (CI 97.75–99.99%). Among 10 screws graded as inaccurate on CT, 9 were graded as such on the CBCT, giving a sensitivity of 90.0% (CI 55.5–99.75%). Among the 250 screws graded as accurate on CT, 244 were graded as such on the CBCT, giving a specificity of 97.6% (CI 94.85–99.11%).

**Conclusions:**

CBCT, performed intraoperatively with the Allura ARSN system, is comparable and non-inferior to a conventional postoperative CT scan for ruling out misplaced pedicle screws in spinal deformity cases, eliminating the need for a postoperative CT.

**Key Points:**

• *Intraoperative cone beam computed tomography (CT) using the Allura ARSN is comparable with conventional CT for ruling out pedicle screw breaches after spinal fixation surgery.*

• *Intraoperative cone beam computed tomography can be used to assess need for revisions of pedicle screws making routine postoperative CT scans unnecessary.*

• *Using cone beam computed tomography, the specificity was 97.6% and the sensitivity was 90% for detecting pedicle screw breaches and the negative predictive value for ruling out a pedicle screw breach was 99.6%.*

## Introduction

Pedicle screw placement, a crucial step in all posterior spinal fixation surgeries, carries an inherent risk due to the close anatomical relationship between the pedicles and important neurovascular structures [1, 2]. A recent meta-analysis demonstrated that 3.3% of free-hand (FH) spinal fixation surgeries led to a postoperative revision surgery to correct misplaced screws [3]. If these incorrect placements of screws could be prevented, patient morbidity would be reduced as well as costs amounting to $23,865–$32,915 per revision surgery [3, 4].

To increase pedicle screw accuracy and minimize the misplacement risk, technical aids such as fluoroscopy, computer-assisted navigation, surgical robots, and neurophysiological monitoring can be used [5–7]. Probing the pilot hole before placing the pedicle screw is commonly part of the surgical routine [8], but cannot reveal a breach occurring after pedicle screw insertion [9]. Irrespective of surgical method, however, the use of intraoperative radiology to evaluate final screw positions can help the surgeon to avoid complications related to screw misplacements and minimize the need for reoperation [10, 11].

Several techniques for detecting pedicle breaches have been described. When the procedures are performed using the FH technique, a mobile C-arm is typically used to acquire 2D fluoroscopic images to intraoperatively verify screw placement [12]. However, this type of imaging cannot create axial views and therefore mediolateral breaches can only be identified through an anteroposterior projection view. The sensitivity of 2D radiographic imaging to identify screws breaching mediolaterally in non-deformity cases is 74% [13] and decreases to 52% in deformity cases [14]. Other intraoperative imaging options include intraoperative CT [15] and intraoperative 3D fluoroscopy [16, 17], both of which have shown promising results in detecting misplaced pedicle screws, thereby potentially reducing the rate of revision surgery.

Cone beam computed tomography (CBCT) was initially developed for use in dentistry, interventional radiology, and orthopedics [18]. The cone beam geometry was developed as an alternative to conventional CT using either fan-beam or spiral-scan geometries, to enable fast data acquisition of the entire field of view (FOV). Imaging is performed by a rotating gantry incorporating an x-ray source and a detector. In contrast to conventional CT which uses a fan-shaped x-ray beam in a helical progression, a divergent pyramidal- or cone-shaped radiation source is directed towards the area of interest and onto an x-ray detector on the opposite side. Using a complete or partial arc, multiple (from 150 to more than 600) sequential planar projection images of the FOV are acquired during the rotation. Since CBCT exposure incorporates the entire FOV, one rotational sequence of the gantry is enough to acquire required data for image reconstruction. A shorter examination time results in increased image sharpness and reduced distortion caused by patient movement, and increased x-ray tube efficiency. A disadvantage, however, is a limitation in image quality related to noise and contrast resolution since large amounts of scattered radiation are detected [19, 20].

An augmented reality surgical navigation (ARSN) system for spine surgery has previously been evaluated in a prospective cohort study and it was demonstrated that it can be used for accurate pedicle screw placement [21, 22]. The ARSN system relies on an intraoperative CBCT (Allura Xper FD20, Philips Healthcare) for preoperative planning as well as postoperative confirmation of screw positions. In brief, a ceiling-mounted robotic C-arm with integrated video cameras within the flat x-ray detector frame is used for imaging and subsequent augmented reality navigation in a hybrid operating room. Multiple adhesive skin markers form the basis for a virtual reference grid to track the patient’s position. The system uses an inherent software providing automatic spine segmentation and pedicle identification, utilized for planning of pedicle screw trajectories and surgical navigation as well as intraoperative confirmation of results [23, 24]. In this study, we retrospectively compared the screw grading on intraoperative CBCT scans to postoperative CT scans of the same patients to evaluate whether the image quality of CBCT is adequate to rule out pedicle breach.

## Methods

This retrospective study was performed as part of a prospective cohort study approved by the institutional review board (2018/1490-31 and 2015/1640-31/2). The prospective enrollment was performed consecutively and all patients eligible for deformity correction spine surgery over the age of 16 were included after signed informed consent was signed. Authors without conflicts of interest had full control of inclusion of data and information submitted for publication.

### Patient characteristics

In total, 20 patients (260 pedicle screws; overall mean age 30.5 years; 11 women and 9 men) undergoing spinal fixation surgery including placement of pedicle screws using the augmented reality surgical navigation (ARSN) system were included [22]. There were 13 scoliosis (9 idiopathic, 2 neuromuscular, and 2 degenerative), 2 kyphosis (1 post fracture and 1 Scheuermann’s), 3 lumbar spondylolisthesis, 1 lumbar spinal stenosis, and 1 lumbar degenerative disk disease. The pre- and postoperative Cobb angles for the 15 deformity cases were 55 ± 16 and 23 ± 13°, respectively [22, 25, 26]. Of the 260 assessed screws, 166 (64%) were placed in the thoracic and 94 (36%) in the lumbosacral spine. The average pedicle width-to-screw size ratio for the whole cohort was 1.3 ± 0.7 and 1.1 ± 0.5 in the 15 deformity cases; The ratio was 1.4 ± 0.8, 1.0 ± 0.3, and 0.8 ± 0.2 for screws rated as grade 0, 1, and 2, respectively [22, 26].

### Surgical technique

The detailed workflow of the surgical setup and system has been published previously [22, 24, 27]. All surgeries were performed in a hybrid operating room (OR) with intraoperative CBCT capability and adapted for spine surgery. In short, all patients were operated on in the prone position. After draping and exposure of the relevant levels, adhesive skin markers were used for patient position tracking. An initial CBCT was acquired, and a surgical plan adapted for the intended pedicle screws using the ARSN software [23]. For every pedicle, an awl followed by a gearshift or power drill was used to create a pilot hole, before placing the pedicle screw using a screwdriver. After placement of all pedicle screws, a confirmatory CBCT was performed to evaluate all screw positions. If needed, according to this CBCT, screws were revised [22].

### CBCT imaging technique

The system consists in part of a ceiling-mounted robotic C-arm (Allura Xper FD20, Philips Healthcare) used for both the initial CBCT scan, for planning and navigation features, as well as the confirmatory CBCT scan at the end of the surgery. Fluoroscopy imaging in 2D with 3.75 x-ray pulses/second was used for spinal level identification and iso-centering of the region of interest prior to 3D CBCT imaging. There were three types of CBCT protocols available: small (12.6 × 12.6 cm^2^), medium (17.3 × 17.3 cm^2^), and large (25.2 × 19.5 cm^2^) detector FOV, as has previously been described [27]. The small FOV protocol used 482 x-ray pulses to generate the CBCT image (the other protocols used 302 pulses), providing an improved image quality at the expense of an increased patient radiation exposure. The three protocols typically include 4 to 7, 5 to 9, and 6 to 10 spinal levels, respectively. During the first 10 procedures, a total of 38 CBCT acquisitions were performed (19 small and 19 medium FOV). During the final 10 procedures, a total of 39 CBCT acquisitions were performed (1 small, 21 medium, and 17 large FOV) [24]. All three protocols had a fixed tube kilovoltage of 120 kV, and a tube current time product ranging from 50 to 325 mAs, modulated by an automatic dose rate control to achieve a similar image quality independent of the patient size. Beam spectral filtration of 0.4 mm copper (Cu) and 1 mm aluminum (Al) was used for all protocols, in addition to 3.5 mm Al inherent x-ray tube filtration yielding 2.4 to 3.1% noise levels based on the ACR 464 CT phantom [27, 28]. For image acquisition, the C-arm rotated 180° in 8–10 s, and in an additional 15 s, a 3D reconstructed volume with 0.5 mm voxel size was displayed at 1 mm thickness in axial and sagittal views with a contrast resolution of 3 to 5 HU [28, 29]. This procedure was performed under temporary apnea in clinical cases [22].

The median total procedure time was 379 min (232–548). The median preparation time from skin incision until before the CBCT acquisition was 107 min (68–174 min), accounting for 28% of the total procedure time. The CBCT acquisition time (for both planning and verification of screw placement) and segmentation of the spinal levels of interest for surgery corresponded to 2% of the total procedure time with a median time of 8 min (range 2–44 min). Total navigation time corresponded to almost 25% of the total procedure time [24]. For verification, the C-arm can be tilted ± 20°, to avoid collinearity between the x-ray beam and the direction of the screws, thus reducing metal artifacts in the 3D images.

For navigation planning and screw placement, a median of 2 [1–4] CBCT acquisitions was performed. An additional 2 [1–4] CBCT acquisitions were performed for verification, replacing postoperative CT. The average AK and DAP per procedure was 159 ± 16 mGy and 31.3 ± 2.8 Gy cm^2^, respectively. The average patient ED was 15.8 ± 1.8 mSv with CBCT contributing to 97 ± 1% of the total procedure ED. The average staff exposure per procedure was 0.21 ± 0.06 mSv [27].

### Screw position assessment

**Fig. 1:**
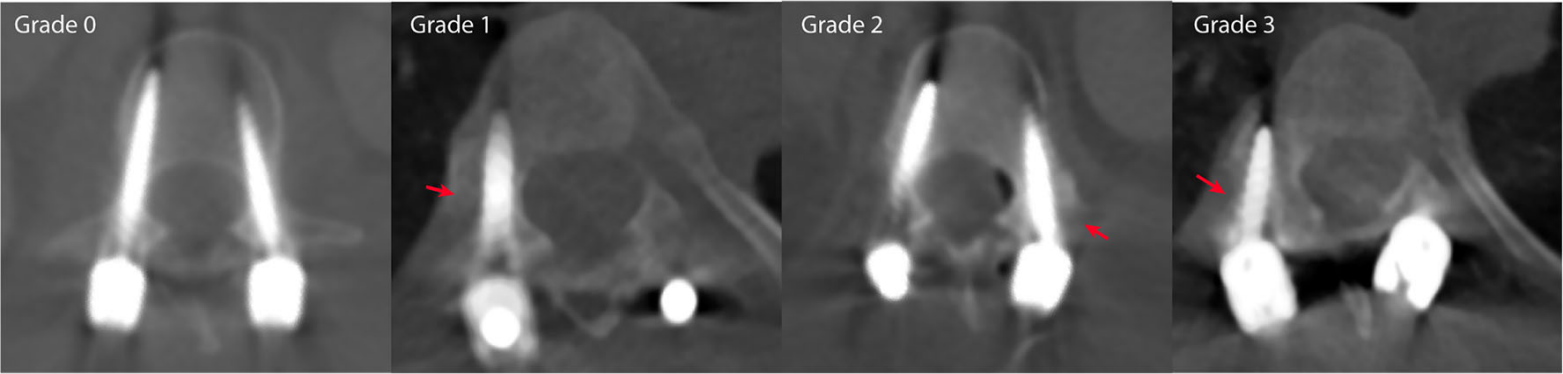
Examples of screws graded as Gertzbein grades 0–3. Red arrows highlight the position of each breach

In addition to the intraoperative CBCT at the end of the surgery, the follow-up protocol included a conventional postoperative follow-up CT at median 12 months (0–21 months) after surgery, as detailed in Table 1. For this study, both the intraoperative CBCT and the postoperative CT were analyzed to determine the presence and degree of pedicle screw perforation outside of the pedicles. Blinded radiological assessments of pedicle screw positions were performed by three independent spine surgeons on both the CBCT and CT scans, and the CT scans served as the standard of reference. All screws were graded according to the Gertzbein grading scale on both the intraoperative CBCT and the postoperative conventional CT scans [30]. Examples of all Gertzbein gradings are seen in Fig. 1, grade 0 (screw within the pedicle without cortical breach), grade 1 (0–2 mm breach, minor perforation including cortical encroachment), grade 2 (> 2–4 mm breach, moderate breach), and grade 3 (> 4 mm breach, severe displacement). Screws graded as 0 or 1 according to the Gertzbein grading scale were considered clinically accurate while grades 2 or 3 were considered clinically inaccurate. Sensitivity, specificity, and negative predictive value were the primary metrics of diagnostic performance.

**Table 1:** Specifications of CT scans used as gold standard

CT scan specification	Median (IQR)
No. of rows	128 (128–256)
Spiral pitch factor	0.98 (0.8–1.38)
Peak tube voltage (kV)	120 (100–120)
Tube current (mA)	92 (54–187)
Slice thickness (mm)	0.65 (0.63–0.75)
Single detector collimation (mm)	0.60 (0.60–0.63)
Rotation speed (s)	0.50 (0.50–0.50)
CTDI_vol_ (mGy)	6.60 (1.68–12.51)
Voxel size	0.71 (0.63–0.75)

*CTDI*_*vol*_ volume computed tomography dose index, *CT* computed tomography, *IQR* interquartile range

### Statistical analysis

Analyses were performed using RStudio (RStudio Team (2016). RStudio: Integrated Development for R. RStudio, Inc.) and R version 3.6.1. Absolute interrater agreement was calculated without corrections, meaning the number of screws where all three raters agreed was divided by the total number of screws [31, 32]. Fleiss’ Kappa was used for calculating reliability of agreement between the 3 raters [33]. Sensitivity, specificity, positive predictive value (PPV), and negative predictive value (NPV) of screw placement assessment based on intraoperative CBCT imaging were computed, using assessment of postoperative CT scans as the gold standard. Values of sensitivity, specificity, PPV, and NPV are presented with their 95% confidence intervals (CI: min%–max%). Confidence intervals for sensitivity, specificity, diagnostic accuracy, and positive and negative predictive values were calculated as described by Collet using R-library “epiR” [34]. ROC analysis was performed using R-library “ROCR” [35].

## Results

**Fig. 2:**
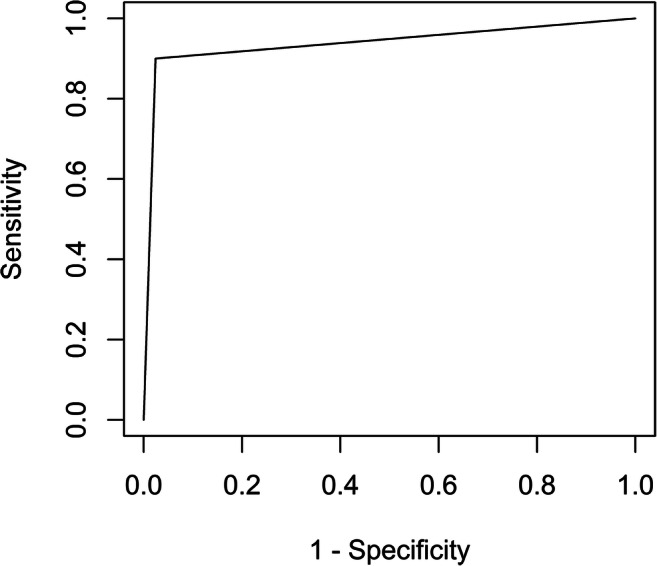
ROC curve for Gertzbein gradings on intraoperative CBCT with postoperative CT as gold standard

In total, 260 pedicle screws were assessed on both intraoperative CBCT and postoperative CT (Table 2). Three screws were identified as severely misplaced on the CBCT and revised intraoperatively and could consequently not be assessed on CT and were excluded from the material. The absolute interrater agreement was 72.7% in the CT group and 63.1% in the CBCT group while Fleiss’ Kappa was 0.48 in the CT group and 0.63 in the CBCT group. Among the 10 (3.8%) screws graded as inaccurate on CT, 9 were graded as such on the CBCT, giving a sensitivity of 90.0% (CI 55.5–99.75%). The single case of false negative screw rating had a Gertzbein grading of 1 according to the CBCT and 2 according to the CT, indicating a borderline case. Among the 250 screws graded as accurate on CT, 244 were graded as such on the CBCT, giving a specificity of 97.6% (CI 94.85–99.11%). In all cases where a screw was rated as incorrect on CBCT but correct on CT, the Gertzbein grading was 2 on CBCT and 1 on CT, indicating borderline cases. Table 3 summarizes the test results and Fig. 2 shows the ROC curve for sensitivity and specificity for detecting inaccurate screws (i.e., Gertzbein grades 2–3).

**Table 2:** Comparison between screw assessment on intraoperative CBCT scans and postoperative CT scans according to the Gertzbein grading scale

		Postoperative CT assessment				
	Grade	0	1	2	3	Total
Intraoperative CBCT assessment	0	*149*	10	0	0	159
	1	32	*53*	1	0	86
	2	0	6	*8*	1	15
	3	0	0	0	*0*	0
	Total	181	69	9	1	260

*CBCT* cone beam computed tomography

Note: Italicized numbers indicate number of agreements between CT and CBCT assessment

**Table 3:** Summary of test statistics for intraoperative CBCT assessment of screws

Parameter	Value	CI (95%)
Sensitivity	90% (9/10)	55–100%
Specificity	98% (244/250)	95–99%
Diagnostic accuracy	97% (253/260)	95–99%
PPV	60% (9/15)	32–84%
NPV	100% (244/245)	98–100%

*CI* confidence interval, *NPV* negative predictive value, *PPV* positive predictive value

The NPV was 99.6% (CI 97.75–99.99%), indicating a high predictive confidence for ruling out pedicle screw breaches in this patient population and setting. The PPV was 60% (CI 32.29–83.66%) indicating a medium predictive confidence for finding pedicle screw breaches. The detailed gradings of all cases where the two methods disagreed are seen in Table 4.

**Table 4:** Screw gradings per reader and modality of cases where CBCT and CT differed in their final screw rating (i.e., correct/incorrect screw rating)

	Reader 1—CBCT	Reader 2—CBCT	Reader 3—CBCT	Reader 1—CT	Reader 2—CT	Reader 3—CT	Pedicle	Follow-up time, CT (months)	Breach direction	Pedicle:screw width ratio
Case: False negative	2	1	1	1	2	2	T9 left	12	Lateral	1.25
Case: False positive #1	2	2	2	1	1	1	T7 left	12	Lateral	0.67
Case: False positive #2	1	2	2	1	2	1	T7 left	0	Lateral	0.78
Case: False positive #3	2	2	2	1	1	1	T11 left	7	Medial	1.44
Case: False positive #4	2	2	2	0	1	1	T10 left	8	Lateral	1.20
Case: False positive #5	2	2	2	1	1	1	T9 right	9	Lateral	0.89
Case: False positive #6	2	1	2	1	1	1	T10 right	9	Lateral	0.84

*CBCT* cone beam computed tomography, *CT* computed tomography

## Discussion

Traditionally, fluoroscopy has been the method of choice for image guidance when placing pedicle screws using the free-hand technique. Most modern surgical navigation technologies, however, use a 3D imaging modality for intraoperative guidance [11]. While surgical navigation enables the surgeon to place screws safely and with higher accuracy than the conventional free-hand method [6], anatomical variations, bone quality, and pedicle geometry may affect the placement and result in a number of pedicle screws needing revision. Detecting these screws intraoperatively would allow immediate repositioning to minimize misplacement-related injuries and reduce revision surgery rates.

Compared with fluoroscopy, the patient radiation dose may increase with a 3D CBCT acquisition. However, this dose is on average less than that from a single-spine CT examination [36]. On the other hand, intraoperative 3D CBCT imaging may potentially prevent repeat surgeries and reduce the amount of follow-up imaging needed, thereby reducing the cumulative patient radiation exposure. This is particularly important in young adult scoliosis patients who are more radio-sensitive than older patient groups and have a higher overall long-term cancer mortality [37, 38]. CBCT scanners are a heterogenous group differing in patient radiation dose and image quality. The hybrid OR-based system used in this study exceeds the performance of common mobile systems regarding Hounsfield unit accuracy, noise, and uniformity due to added x-ray filtration and automatic exposure control [28]. Replacing the postoperative CT scan by a good-quality intraoperative CBCT may therefore have multiple benefits.

**Fig. 3:**
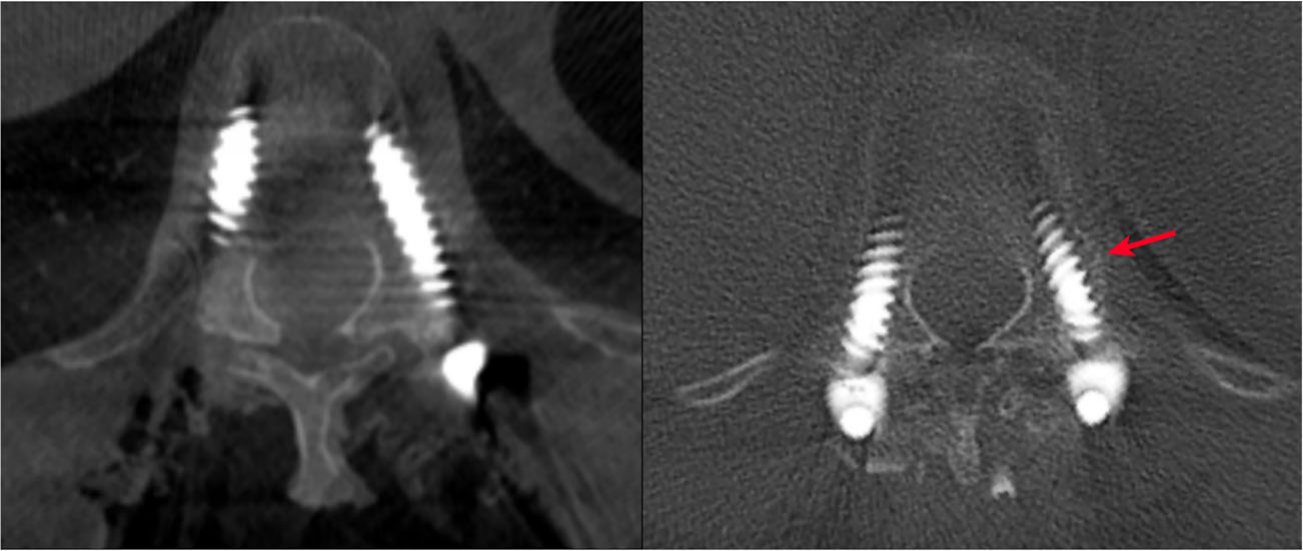
Comparison between CBCT (left) and CT images (right) depicting suspected bone overgrowth between the two images were taken. Red arrow highlights suspected bone overgrowth

Thus, correct intraoperative assessment of pedicle screw placement is of great importance. In this study, we have shown that the use of intraoperative CBCT to assess pedicle breach in a consecutively enrolled cohort of deformity cases has a NPV of 99.6%. This means that if the CBCT does not indicate that a breach has occurred, the surgeon can be highly confident that this is in fact true. The PPV of 60% indicates that even though a screw looks misplaced on CBCT, this might not be the case on a postoperative CT. In fact, only 3 screws in our series were intraoperatively revised [22]. When looking at the cases where CT showed a lower Gertzbein grading than the CBCT, we could conclude that part of the difference may be explained by bone remodeling and bone growth around the pedicle screw between the time of intraoperative CBCT and follow-up CT, as demonstrated in Fig. 3.

Our results are in accordance with results obtained by Cordemans et al, comparing CBCT (Artis Zeego II) to conventional CT scans performed on average 3 months after surgery due to different clinical requirements [39]. Grading was performed by the two operating surgeons separately and in a joint session to reach consensus. This approach may bias the final grading, and hence, we did not attempt to reach consensus between our independent raters. Only 2% of their cases were scoliotic, compared with our 65%. Dysplastic pedicles, as seen in spinal deformities, may be more difficult to assess radiologically, especially if screw diameters are large compared with pedicle width. In our case with a pedicle-to-screw diameter ratio of 1.1 ± 0.5, distinguishing between grades 1 and 2 was often a delicate matter. In a similar study Nevzati et al, using Allura FD20 without ARSN, demonstrated that the sensitivity and specificity for breach detection was higher for severe displacement compared with perfectly placed screws [10]. A summary of similar comparative studies is presented in Table 5.

**Table 5:** Overview of studies and their test statistics for intraoperative screw position assessment

Study	Year	No. of screws	Device	Compared to	Sensitivity	Specificity	PPV	NPV
Rao [40]	2002	155	CT	Open dissection	86	85	95	62
Santos [17]	2012	416	O-arm	Open dissection	76	71	74	72
Garber [16]	2012	73	O-arm	CT	85	97	82	98
Nevzati [10]	2017	241	Allura FD20	CT	71–92	77–99		
Cordemans [39]	2017	348	Artis Zeego II	CT	77	98	86	96
Current study	2019	260	Allura ARSN	CT	90	98	60	100

*ARSN* augmented reality surgical navigation, *CBCT* cone beam computed tomography, *CT* computed tomography, *NPV* negative predictive value, *PPV* positive predictive value

Compared with previous studies, we have a higher sensitivity, specificity, and NPV while the PPV is lower in our study. This could in part be explained by a lower proportion of inaccurate pedicle screws in our material, reflected in the high NPV but low PPV. In part, this is explained by intraoperative revision of obviously misplaced screws identified by CBCT, removing them from the statistical analysis. In total, 3 screws were revised intraoperatively. Assuming that they could also be correctly identified on postoperative CT would increase the PPV to 0.67. In any case, this study was performed on an unbiased patient material consisting of consecutively enrolled patients at our institution; the NPV and PPV reflect the clinical reality of using the system for the intended patient group. Our test statistics would therefore be expected to be valid for spine deformity cases at similar tertiary care institutions.

A hybrid OR can improve facility utilization by covering many procedures—from endovascular to minimally invasive or open surgery—and enable exploration of new procedures that leverage intraoperative high-quality imaging and high level of device integration [41]. The results of this study indicate that the intraoperative CBCT imaging generated in a hybrid OR is of sufficient quality, comparable with that of conventional CT, to reliably identify pedicle screw misplacements. Extending these findings to other uses in the hybrid OR, we suggest that intraoperative CBCT may replace the postoperative CT in many spine and interventional procedures requiring accurate 3D imaging.

### Limitations of this study

One of the main limitations of this study is the use of conventional CT as the gold standard. We justified this approach with CT being the currently used standard for evaluating pedicle screw placement [40]. However, the image quality of CT scans and CBCT scans varies depending on manufacturer and used dose protocol, indicating that some CBCT protocols might provide better image quality than CT and vice versa. Consequently, we used CT scans performed as part of the normal clinical routine to reflect the clinical reality. A drawback with this approach is that the image quality of the postoperative CT scans was variable, in part due to several different low-dose protocols applied in the routine care, as detailed in Table 1. In some cases, we subjectively found the image quality to be better in the Allura CBCT scans than the conventional CT scans. Data on radiation exposure for staff and patient using the ARSN system has been previously reported [27].

The CT scans included in this study were performed when clinically advised and therefore varied in time after surgery with a median follow-up of 12 months (0–21 months) postoperatively. It could be argued that scans obtained a long time after the operation would not necessarily be identical to the intraoperative imaging due to bone remodeling including both bone resorption and new bone formation around the pedicle screws. If anything, this would work to attenuate the findings of this study and not exaggerate them. One example of this was where we suspected bone formation around pedicle screws that were judged as false positives in our study, as highlighted in Fig. 3, since CBCT indicated breach but CT did not.

Another limitation could be that the intraoperative CBCT was obtained after pedicle screw placement but prior to rod placement and deformity correction. In that way, breaches resulting from pressure applied on pedicle screws due to rod placement and correction forces might be missed. However, we could not identify any clinically relevant effect of this possible phenomenon in our material.

## Conclusion

Intraoperative CBCT, performed in a hybrid OR equipped with the Allura ARSN system, is reliable for ruling out pedicle screw breaches and can be used for intraoperative breach detection and revision, making routine postoperative CT scans unnecessary.

## Abbreviations

ARSN: Augmented reality surgical navigation
CBCT: Cone beam computed tomography
CI: Confidence interval
CT: Computed tomography
FH: Free hand
FOV: Field of view
NPV: Negative predictive value
OR: Operating room
PPV: Positive predictive value
